# Results From a Psychometric Validation Study: Patients With Irritable Bowel Syndrome Report Higher Symptom Burden Using End-of-Day Vs Real-Time Assessment

**DOI:** 10.14309/ajg.0000000000003091

**Published:** 2024-09-23

**Authors:** Michelle Bosman, Lisa Vork, Daisy Jonkers, Johanna Snijkers, Rabia Topan, Qasim Aziz, Irina Midenfjord, Magnus Simren, Ad Masclee, ESM study group, Daniel Keszthelyi

**Affiliations:** 1 Department of Gastroenterology-Hepatology, NUTRIM School of Nutrition and Translational Research in Metabolism, Maastricht University Medical Center, Maastricht, the Netherlands;; 2 Wingate Institute of Neurogastroenterology, Centre for Neuroscience and Trauma, Blizard Institute, Barts and the London School of Medicine & Dentistry, Queen Mary University of London, London, United Kingdom;; 3 Department of Internal Medicine, Institute of Medicine, Sahlgrenska Academy, University of Gothenburg, Gothenburg, Sweden.

**Keywords:** irritable bowel syndrome, experience sampling method, patient-reported outcome, psychometric evaluation

## Abstract

**INTRODUCTION:**

Real-time assessment of gastrointestinal (GI) symptoms in irritable bowel syndrome (IBS) using the experience sampling method (ESM) is suggested as a more appropriate approach than currently used end-of-day or end-of-week reports. This psychometric evaluation study assesses the validity and reliability of a previously developed ESM-based patient-reported outcome measure (PROM) for real-time GI symptom assessment in IBS.

**METHODS:**

This multicenter validation study included 230 Rome IV patients with IBS (80% female; mean age 41.2 years) in 3 European countries. Patients completed the electronic ESM-PROM (up to 10 random moments daily, with a weekly minimum completion rate of 33%) and an end-of-day symptom diary for 7 consecutive days. End-of-week questionnaires (Gastrointestinal Symptom Rating Scale for IBS, IBS Severity Scoring System, Patient Health Questionnaire-9, and Generalized Anxiety Disorder-7) were completed at the end of the 7-day period.

**RESULTS:**

The ESM assessment had a mean completion rate of 71%. Strong and significant correlations (0.651–0.956) with moderate-to-good consistency (intra-class correlation coefficients 0.580–0.779) were observed between ESM and end-of-day scores. However, end-of-day scores were significantly higher (Δ0.790–1.758, *P* < 0.001) than mean daily ESM scores. Differences with end-of-week scores were more pronounced, with weaker correlations (Pearson's *r* 0.393–0.802). ESM-PROM exhibited moderate-to-good internal consistency (Cronbach's α 0.585–0.887) across 5 symptom domains. First and second half-week scores demonstrated good-to-excellent consistency (intraclass correlation coefficients 0.871–0.958).

**DISCUSSION:**

Psychometric evaluation demonstrated strong validity and reliability of the ESM-PROM for real-time GI symptom assessment in IBS. In addition, the ESM-PROM provides a precise and reliable ascertainment of individual symptom pattern and trigger interactions, without the bias of peak reporting when compared with retrospective methods. This highlights its potential as a valuable tool for personalized healthcare in monitoring disease course and treatment response in patients with IBS.

## Graphical abstract

**Figure:**
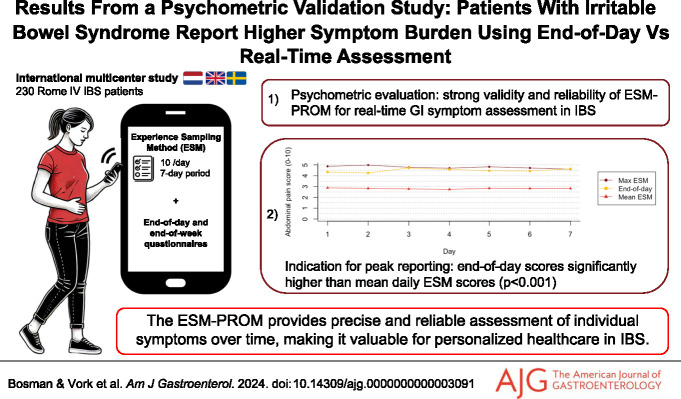

## INTRODUCTION

Irritable bowel syndrome (IBS), a disorder of gut-brain interaction (DGBI), is characterized by abdominal pain and altered bowel habits (1). The pathophysiology of IBS is multifactorial and not completely understood, and reliable biomarkers are lacking. Consequently, diagnosis of IBS is symptom-based (i.e., Rome IV criteria), and monitoring of disease course and evaluation of treatment efficacy are primarily focused on subjective reporting of symptom severity. In addition, the heterogeneous IBS patient population is characterized by between-subject and within-subject variations in symptoms (2,3). Therefore, accurate patient-reported symptomatology monitoring is of utmost importance in this patient population (3). In clinical trials, current regulatory guidelines recommend using a composite primary endpoint that assesses abdominal pain and stool changes according to Rome IV criteria, using retrospective end-of-day diaries or end-of-week questionnaires (4,5). Such retrospective assessment methods, however, are often affected by psychological biases, including recall bias related to patients' autobiographical memory and ecological bias because of contextual and psychological influences (6). Moreover, these assessments are not able to monitor daily symptom fluctuations (6,7). Consequently, retrospective measurement leads to a distorted and inaccurate representation of the patients' symptomatology not closely reflecting the actual symptoms experience.

Recent research has focused on the experience sampling method (ESM) as a real-time symptom assessment approach to overcome the shortcomings of retrospective end-of day or end-of week measurements. ESM collects in-the-moment assessments of the participant's current state and real-life environment, randomly and repeatedly during the day over several consecutive days, using electronic devices. This method results in a registration of real-life symptom patterns, including moment-to-moment variability, while minimizing recall and ecological bias and improving compliance rates (6,7).

ESM has been applied for symptom assessment in various disorders, including somatic, psychiatric, and chronic pain disorders (7–11), and is gaining attention in the field of DGBI as well (12–15). Recently, we developed an ESM-based patient-reported outcome measure (PROM) specific for patients with IBS (16). Content validity was assured, and the tool was cross-culturally adapted for use in the Netherlands, Belgium (Flemish), Sweden, the United Kingdom, and the United States. (17). It has proven to be a valuable tool for offering detailed insights into symptom patterns and its potential in personalizing treatment strategies (18,19). However, further validation is needed to assess the psychometric properties of this ESM-PROM, including its reliability and validity, for symptom assessment in IBS populations (20). Therefore, the current psychometric evaluation study aims to evaluate the validity and reliability of the previously developed ESM-PROM for symptom assessment in an IBS population, with the potential to serve as a valuable instrument to effectively monitor disease course and treatment response.

## METHODS

### Study population and setting

This psychometric evaluation study includes prospective data from 3 studies on IBS, all using the ESM-PROM tool, grouped into 2 cohorts: (i) an international multicenter study specifically designed for validating the ESM-PROM tool in IBS (MEASuRE study), combined with the baseline data from an observational single-group, open-label study of linaclotide in IBS-C at Maastricht University Medical Center (UMC) + using the ESM-PROM tool, as the data collection in these 2 studies was identical (*cohort 1*), and (ii) data collected at Maastricht UMC + as a part of a large European multicenter observational study aimed on enhancing the understanding of IBS using among others, the ESM-PROM tool (DISCOvERIE study; *cohort 2*). There was no overlap of patients between the cohorts. Detailed information on the cohorts is given in the Supplementary Digital Content (see Supplementary Appendix 1, http://links.lww.com/AJG/D412).

Patient recruitment took place between 2017 and 2022 at multiple secondary/tertiary hospitals across Europe (Maastricht UMC+, the Netherlands; Sahlgrenska University Hospital, Sweden; Royal London Hospital, United Kingdom). All patients met the Rome IV criteria, confirmed in a face-to-face interview by a trained investigator. All 3 study protocols were approved by the Committee of Ethics in the participating centers and were executed according to the revised Declaration of Helsinki (64th World Medical Association General Assembly, Fortaleza, Brazil; October 2013) and International Council on Harmonisation of Technical Requirements for Registration of Pharmaceuticals for Human Use good clinical practices guidelines. The studies were registered in the US National Library of Medicine: NCT02880722 and NCT03336034 (cohort 1). All participants gave written informed consent before participation. All authors had access to the study data and reviewed and approved the final manuscript.

### Data collection

**Figure 1.**
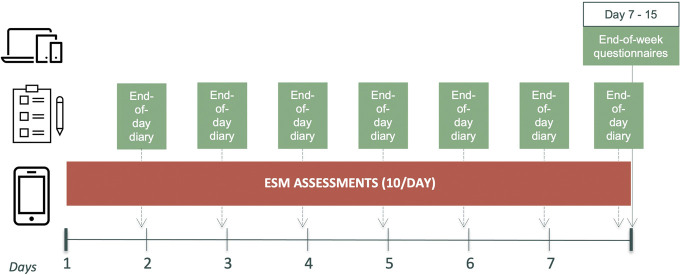
Data collection during a period with ESM assessments, end-of-day diaries, and end-of-week questionnaires. ESM, experience sampling method.

Data collection is visualized in Figure 1. All participants filled in demographic questionnaires and completed ESM assessments for 7 consecutive days. In cohort 1, participants also completed paper end-of-day symptom diaries during this period. After this 7-day period, all participants completed validated retrospective symptom questionnaires. Participants initiated the 7-day period on different days of the week (i.e., start day ranging from Monday to Sunday).

#### ESM.

A digital application, developed specifically for the use of ESM in patients with IBS (17), was installed and activated on the participants' smartphones for a 7-day period. The ESM application was set to send out both auditory and written signals at random moments, occurring 10 times each day between 07:30 am and 10:30 pm, with at least 15 minutes between consecutive signals. After each signal, participants had a 10-minute window to complete the ESM questionnaire. Questionnaires left unanswered after this 10-minute window were considered as missing data. Participants were instructed to complete the real-time questionnaires as often as possible each day, responding promptly after each signal, but to skip questionnaires when completion was considered impossible at the time of the signaling (e.g., while driving a car).

**Figure 2.**
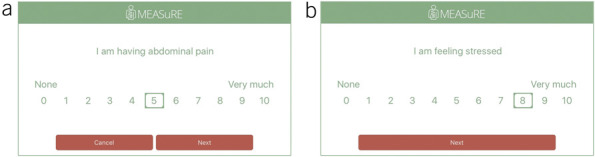
Two examples of questions as asked in the ESM-PROM application (**a**: assessment of abdominal pain and **b**: assessment of feeling stressed). ESM, experience sampling method; PROM, patient-reported outcome measure.

The development of this ESM questionnaire has been described previously (17). In short, the ESM questionnaire contains items assessing 5 domains: physical status (including abdominal pain, other GI [gastrointestinal] symptoms, and general physical complaints), defecation, mood and psychological factors, context and environment, and nutrition and drug use (Figure 2 and see Supplementary Figure 1, http://links.lww.com/AJG/D412). Participants rated all questions on an 11-point Numeric Rating Scale (0 = *not at all* to 10 = *very severely*), in the same order of questions at every assessment moment.

#### End-of-day diary.

In cohort 1, an end-of-day symptom diary was used to evaluate daily GI symptom severity over 7 consecutive days. GI symptoms (i.e., abdominal pain, abdominal discomfort, abdominal bloating, belching, flatulence, constipation, diarrhea, and urge to defecate) were scored using an 11-point Numeric Rating Scale (0 = *not at all* to 10 = *very severely*) at the end of each day. In addition, stool consistency for each bowel habit was recorded during the day using the Bristol Stool Chart (4). For means of comparison between conventional end-of-day diaries and the newly developed electronic, real-time ESM, the symptom diary was completed on paper.

#### End-of-week questionnaires.

After the 7-day ESM period, validated questionnaires were completed digitally. In cohort 1, the questionnaires were completed on the seventh day, whereas in cohort 2, they were completed within 8 days after the 7-day ESM period. This ensured that the recall period of the questionnaires (i.e., 14 days) overlapped with the 7-day ESM period. Severity of IBS symptoms was assessed by using the Gastrointestinal Symptom Rating Scale for Irritable Bowel Syndrome (IBS) (21) in cohort 1 and the IBS Severity Scoring System (22) in all participants. Comorbid symptoms of anxiety and depression were assessed in all participants using both the Generalized Anxiety Disorder-7 (23) and the Patient Health Questionnaire-9 (24), respectively. Detailed information on the questionnaires is given in the Supplementary Digital Content (see Supplementary Appendix 2, http://links.lww.com/AJG/D412).

### Statistical analysis

Detailed information on the statistical analysis is given in the Supplementary Digital Content (see Supplementary Appendix 3, http://links.lww.com/AJG/D412). All analyses were performed using R Statistical Software (v4.2.2; R Core Team [2022]). Participants who completed at least 23 of 70 assessments on ESM were included in the analysis; 11 participants were excluded for not meeting this completion rate (25,26). Differences in baseline characteristics were tested using an analysis of variance with *post hoc* Bonferroni correction for continuous variables and χ^2^ test for categorical variables. In addition, correction for multiple testing was applied using the Bonferroni correction (*P* ≤ 0.007 was considered statistically significant).

Concurrent validity was assessed by comparing ESM scores to end-of-day diary scores in cohort 1 and to end-of-week questionnaire scores in all participants. Associations between end-of-day scores and mean ESM scores (i.e., on day level, combining all repeated measurements of all participants for the concerning day) were tested using linear mixed-effects models, and by calculating intraclass correlation coefficients (ICCs). ICC values between 0.5 and 0.75 are considered moderate, and values above 0.75 are considered good (27,28). Differences between the end-of-day and mean/max ESM scores were tested using intercept-only linear mixed-effects models with the delta scores (i.e., difference between end-of-day diary and ESM) as the dependent variable. Both linear mixed-effects models were corrected for repeated measures by using an autoregression (AR1) covariate structure. ESM measures were averaged per participant to compare ESM scores with end-of-week questionnaire scores. Differences were tested using paired-samples *t* tests. Pearson correlations were calculated, considering values above 0.70 as strong (29). If necessary, ESM scores or end-of-week questionnaire scores were rescaled (see Supplementary Appendix 3, http://links.lww.com/AJG/D412).

Internal consistency and test-retest reliability were assessed in all participants. The internal consistency of the ESM-PROM was evaluated by dividing the items into 5 domains and calculating Cronbach's alpha coefficient per domain. Cronbach's alpha values of 0.7–0.9 are considered to reflect good internal consistency (28). Test-retest reliability was evaluated comparing the mean scores for the first half-week (i.e., days 1, 2, and 3) and second half-week (i.e., days 5, 6, and 7) with paired *t* tests and ICC, considering values between 0.75 and 0.90 as moderate and values greater than 0.9 as excellent (27,28).

#### Sample size.

According to the United States Food and Drug Administration-PROM Expert Group, sample sizes for testing reliability and validity of PROMs should include at least 200 cases, ideally derived from multiple samples (20).

## RESULTS

In this psychometric evaluation study, a total of 230 patients (mean age 41.2 years [95% confidence interval 39.3–43.1]; 80.4% females) were included in 3 European countries (the Netherlands: 117; Sweden: 34; and United Kingdom: 79). Baseline characteristics per cohort are presented in Table 1. Patients from cohort 2 were significantly older and had significantly lower levels of university education than those in cohort 1. Moreover, baseline characteristics were comparable between the countries, except for age, with patients from the United Kingdom being significantly younger than those from the Netherlands. In addition, patients in the Netherlands had significantly lower levels of university education compared with those in the United Kingdom and Sweden (see Supplementary Table 1, http://links.lww.com/AJG/D412).

**Table 1.**
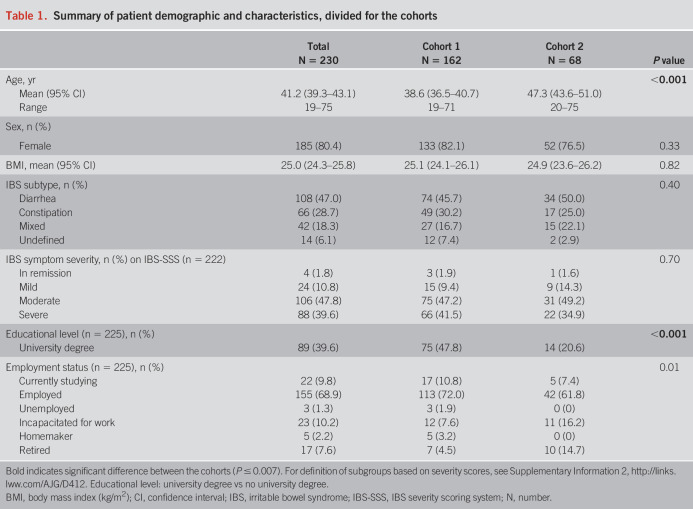
Summary of patient demographic and characteristics, divided for the cohorts

Completion rate of the ESM assessments was 70.6%, corresponding with a mean number of completed assessments of 49.4 (±10.4) out of a total of 70 assessments per participant over the 7-day period. Most participants completed between 51 and 60 assessments, with the lowest number of completed assessments per participant being 23 and the highest number being 69 (see Supplementary Figure 2, http://links.lww.com/AJG/D412). This compliance remained consistent throughout the week, with an average of 7.5–7.9 of a total of 10 assessments each day, independent of the day of the week (i.e., weekday vs weekend) (see Supplementary Table 2 and 3, http://links.lww.com/AJG/D412). In addition, response rates did not fluctuate during the day, except for a slightly lower completion rate at the first assessment of the day (see Supplementary Table 4, http://links.lww.com/AJG/D412). The completion rates for the end-of-week questionnaires were at least 97%, whereas the end-of-day diaries were completed in 91% of the included ESM patients, with less than 1% of diary days missing.

### Concurrent validity

#### End-of-day scores compared with ESM scores.

GI symptoms scored in both ESM and the end-of-day diary were abdominal pain, belching, bloating, flatulence, and urge to defecate. The associations between the mean ESM scores and the corresponding end-of-day scores were all strong and significant (*P* < 0.001), with high correlation coefficients all above 0.815, except for flatulence (Table 2). Furthermore, the ICCs showed moderate-to-good level of agreement between the ESM assessments and the end-of-day diary, with strong consistency for abdominal pain and bloating, and moderately strong consistency for belching, flatulence, and urge to defecate (ranging from 0.580 to 0.779, Table 2).

**Table 2.**
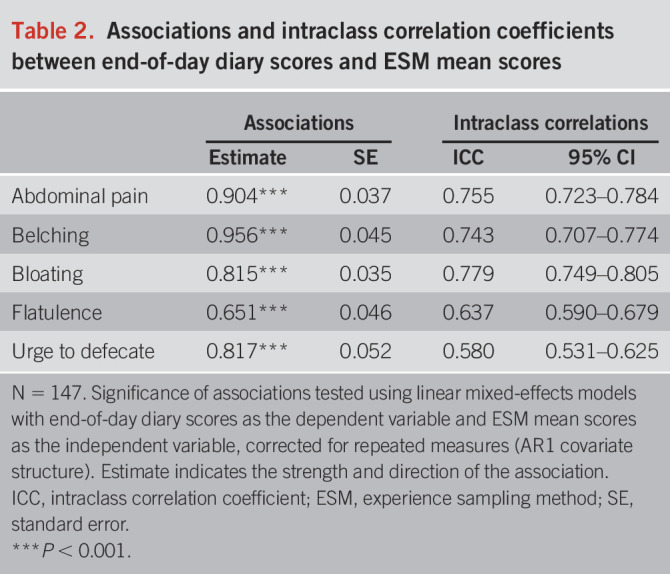
Associations and intraclass correlation coefficients between end-of-day diary scores and ESM mean scores

**Figure 3.**
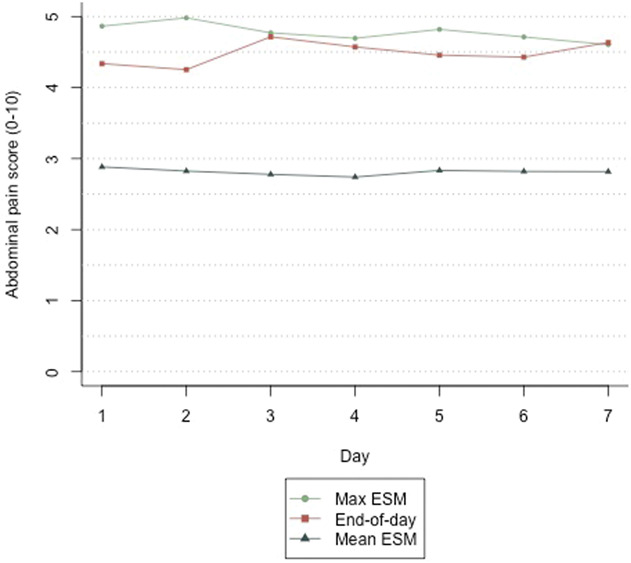
Daily end-of-day diary scores and mean and maximum ESM scores for abdominal pain (on an 11-point NRS). Differences tested using linear mixed-effects models are shown in Table 3 (N = 162). ESM, experience sampling method; NRS, numeric rating scale.

As shown in Table 3, significant differences (*P* < 0.001) were observed in all GI symptoms between end-of-day diary and both the mean and maximum ESM scores, ranging from Δ0.790 to Δ1.758 for mean ESM scores and Δ0.379 to Δ0.939 for maximum ESM scores. Moreover, differences between maximum ESM and end-of-day scores were smaller (also illustrated in Figure 3 and see Supplementary Figure 3, http://links.lww.com/AJG/D412), suggesting that participants tend to report peak symptom levels at the end of the day.

**Table 3.**
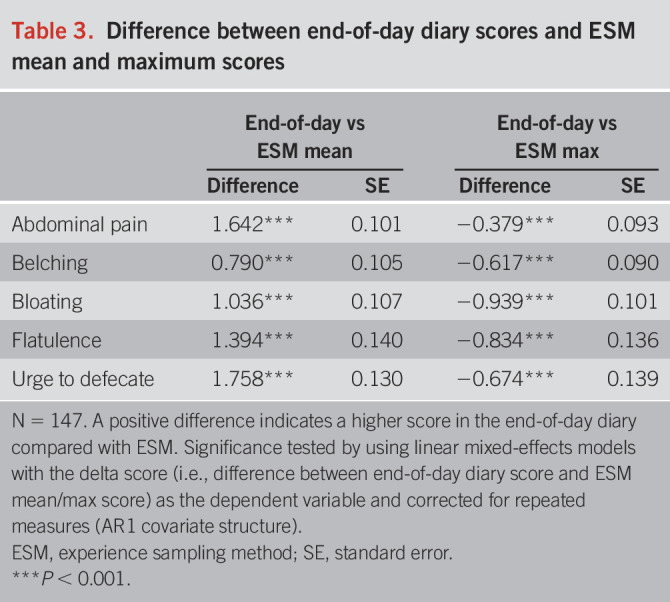
Difference between end-of-day diary scores and ESM mean and maximum scores

#### End-of-week scores compared with ESM.

The comparison and correlation between mean ESM scores and end-of-week questionnaire scores are shown in Table 4. End-of-week Gastrointestinal Symptom Rating Scale scores were significantly higher (*P* < 0.001) than mean ESM scores for abdominal pain, belching, bloating, flatulence, urge to defecate, and heartburn. However, the sensation of feeling full showed significantly higher mean ESM scores (*P* = 0.02) compared with end-of-week scores. Moderate positive correlations (Pearson's *r* range 0.418–0.678) were observed for all symptoms, except heartburn, which showed a strong positive correlation (Pearson's *r* = 0.802). IBS Severity Scoring System scores for abdominal pain and bloating also demonstrated significantly higher scores (*P* < 0.001) compared with corresponding mean ESM scores, with moderate positive correlations (Pearson's *r* = 0.477 and 0.654, respectively). Psychological factors measured by ESM corresponding to Patient Health Questionnaire-9 or Generalized Anxiety Disorder-7 items correlated moderately (Pearson's *r* range 0.472–0.640).

**Table 4.**
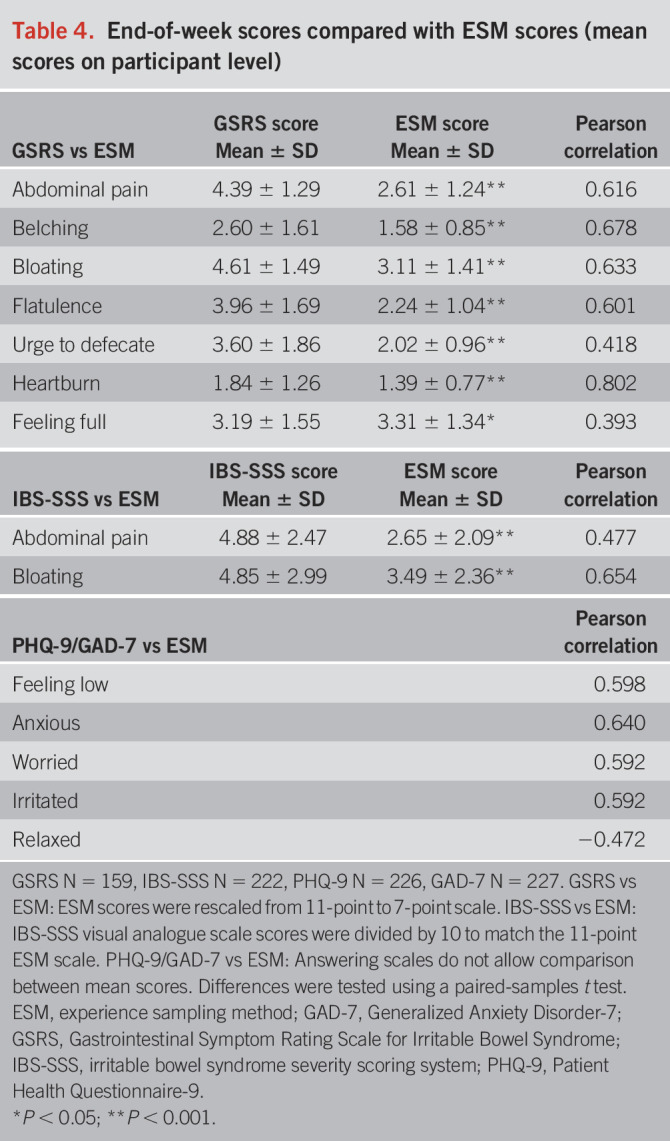
End-of-week scores compared with ESM scores (mean scores on participant level)

### Internal consistency

The internal consistency of 5 symptom domains within the ESM assessments is shown in Table 5. The results showed moderate internal consistency for the upper GI symptoms, physical non-GI symptoms, and positive affect domains (Cronbach's α range 0.585–0.692). Furthermore, the results indicated good internal consistency for the lower GI symptoms and negative affect domains (Cronbach's α = 0.713 and 0.887, respectively).

**Table 5.**
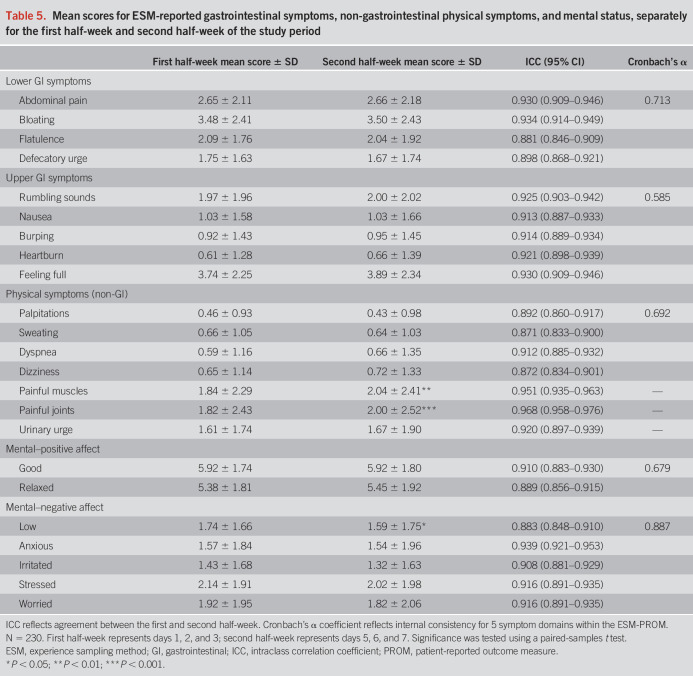
Mean scores for ESM-reported gastrointestinal symptoms, non-gastrointestinal physical symptoms, and mental status, separately for the first half-week and second half-week of the study period

### Test-retest reliability

Mean ESM scores for the first (i.e., day 1, 2, and 3) and second (i.e., day 5, 6, and 7) half-week of the study period are shown in Table 5. Scores did not differ significantly between these 2 periods, except for painful muscles, painful joints, and low affect. ICCs for all symptoms showed good-to-excellent consistency between the 2 periods (ICCs range 0.871–0.958).

## DISCUSSION

In this study with 230 patients with IBS from 3 European countries, we performed a psychometric evaluation of a previously developed PROM based on the ESM principle for momentary contextual symptom assessment in IBS. Content validity and cross-cultural adaptation of this ESM-PROM were described in more detail previously (17). Altogether, the current study demonstrates adequate concurrent validity when comparing ESM scores with end-of-day diaries and validated end-of-week questionnaires, the metrics on which current recommendations for assessing clinical response in IBS are based. Moreover, reliability was considered adequate based on moderate-to-good internal consistency and excellent test-retest reliability.

As IBS is a heterogeneous disorder often influenced by environmental factors and presenting with comorbid psychological and general somatic complaints, it is crucial to develop PROMs that assess GI symptoms along with psychological, somatic, and environmental aspects (3). This was taken into account in the development of this ESM-PROM (17). Before introducing a novel PROM, it is essential to demonstrate validity and reliability, following established guidelines such as those outlined by the US Food and Drug Administration specifically addressing the evaluation of PROMs for IBS (4). We here report the evaluation according to these recommendations that are applicable to the developed electronic ESM-PROM. Assessment of patients' mental state using ESM has been thoroughly described in psychiatric research (30) and items regarding the environmental and social context are not well suitable for psychometric evaluation. For this reason, the primary focus of the current study is on GI symptom monitoring, the key symptoms of IBS.

First, we assessed concurrent validity and found strong significant associations between ESM scores for GI symptoms and corresponding end-of-day scores. Together with moderate-to-good agreement between the assessment methods, this indicates that the ESM-PROM is measuring similar constructs as the end-of-day diary regarding key IBS symptoms. Differences in end-of-day scores compared with mean and maximum ESM scores indicate different levels of symptom reporting during the day assessed by the 2 methods, with the tendency for individuals to retrospectively report higher symptom levels on average throughout the day, compared with the in-the-moment ESM assessments. This apparent overreporting of symptoms aligns with findings of previous ESM studies, indicating peak reporting, i.e., wherein individuals remember the worst pain experience during a day (i.e., recall bias) (6,7,14,16,31). When comparing ESM to end-of-week scores, all end-of-week GI symptom scores were significantly higher and correlations were weaker, except for the sensation of feeling full, which may suggest that this sensation is less memorable or distressing compared with other symptoms when recalled retrospectively. In conclusion, this indicates that the newly developed ESM-PROM effectively measures similar constructs without overreporting of GI symptom scores compared with the recommended end-of-day and end-of-week methods.

Second, we evaluated the internal consistency of the ESM-PROM, showing moderate-to-good internal consistency across all 5 symptom domains. Specifically, the lower GI symptom domain, which addresses the core symptoms of IBS, exhibited good internal consistency, emphasizing its primary importance. Third, we assessed test-retest reliability by comparing and correlating mean scores of the first half-week with mean scores of the second half-week. Despite the expectation of imperfect reliability because of the fluctuating nature of IBS symptoms, the results showed a high level of consistency between the 2 periods. This consistency is likely attributed to smaller within-subject differences compared with between-subject differences and aligns with findings from a previous ESM study in a DGBI (12). For almost all symptoms, no significant differences in mean scores were found between the 2 periods, except for painful muscles, painful joints, and low affect. This was considered less relevant as the tool focuses on GI symptom monitoring and is developed for the screening and scientific investigation of these other non-GI and psychological symptoms in the IBS population.

The ESM-PROM has the potential of providing a more precise and reliable representation of individual symptom patterns and triggers over time compared with the recommended retrospective reporting in IBS clinical trials (4,5). As these retrospective methods are generally accepted, and ESM has a higher time burden, it is worth discussing the benefits ESM provides. The repeated real-time momentary assessments present a detailed perspective on patients' symptomatology in patients' daily lives, ensuring a high level of ecological validity (6,31). The ESM-PROM was used for 7 days; however, the substantial number of repeated measurements enhances the statistical power, increasing sensitivity to detect changes, including symptom formations (i.e., how symptoms impact symptoms) and fluctuations of complaints throughout the day (31). Furthermore, ESM minimizes memory strains and avoids data aggregation, as only the actual moment is assessed repeatedly over time (6). This dynamic approach of ESM results in a comprehensive understanding of individual IBS symptoms in daily life, and facilitates the analysis of interactions among symptoms, such as GI and non-GI symptoms, positive and negative affect, and environmental influences (7,31). Consequently, this approach yields valuable insights into symptom patterns and triggers, for both patients and healthcare professionals, which can be applied in monitoring of disease progression and treatment response in clinical care. In addition, it has the potential to enhance the understanding of IBS pathophysiology in research and pave the road for future interventions, including behavioral interventions. Moreover, the use of the ESM-PROM itself may inherently yield therapeutic benefits by offering patients insights into their symptom patterns and triggers, ultimately enhancing patient self-management. Altogether, this highlights the potential of telemedicine in IBS healthcare, with a rich data set available for utilization in data mining and artificial intelligence.

Nonetheless, it is important to acknowledge some drawbacks associated with ESM. ESM presents a potential patient burden because of its repeated daily assessments, making it more time-consuming than end-of-day and end-of-week reports. In addition, the digital skills of individuals could impact the effectiveness of the tool, and individuals may face challenges in consistently completing repeated daily assessments during the day, particularly with certain types of employment. This potential burden may result in lower compliance; however, in the current study, more than 70% of assessments were completed, exceeding the typically accepted 33% completion rates in other ESM studies (12,25,26). Each electronic tool may face technical issues, such as downloading difficulties or signal blocking, but these were infrequent in our study. Importantly, the application works offline, herewith reducing the impact of such issues on compliance. There is a potential for selection bias as patients may choose to respond to specific questionnaires in the 7-day period. However, in this study, compliance remained consistently high throughout the week and various times of the day, indicating no significant impact from factors such as type of day (i.e., weekdays vs weekends) or participants' moods on assessment completion. Lower compliance occurred in the morning, as expected, aligning with previous studies (26,31). Last, ESM can be associated with induced reactivity or hypervigilance to symptoms because participants can be more conscious of their symptoms during ESM data collection (31). We observed no increase in abdominal pain scores throughout the week, and nearly all scores remained consistent between the first half and the second half of the week, indicating that potential heightened awareness during ESM data collection in this 7-day period does not seem to impact symptom experience over time.

Taking the pros and cons of ESM together, we believe that ESM has some important advantages over traditionally used symptom assessment methods in IBS. However, it may not universally be the most appropriate and feasible method for symptom assessments in all circumstances. The specific research question or clinical situation should be considered when deciding whether or not ESM and which duration of ESM is suitable and of added value (31).

A strength of our study is that development and validation of the evaluated tool were performed in line with recommendations specific to PROMs in IBS (4,5). Furthermore, the large sample size of our international multicenter study, coupled with the extensive number of repeated measurements, significantly enhances the statistical power of our analyses, allowing us to draw firm conclusions regarding the validity and reliability (32). Moreover, using a smartphone application with built-in auditory and visual notifications enhances compliance with the ESM method and eliminates fake compliance.

In addition to the analyses presented here, an important aspect of assessing the quality of a PROM is responsiveness, i.e., the ability of the tool to measure change in symptom scores over time and after treatment (33). However, because of the limited size of the data set available for testing responsiveness in the linaclotide study, we were unable to include this aspect in the current investigation. Our group has previously demonstrated increased sensitivity in measuring treatment response using a similar, handheld-device–based tool for IBS when compared with traditional end-of-day and end-of-week assessments (13). Nevertheless, the responsiveness of this particular ESM-PROM needs to be tested in future studies as well.

In conclusion, the current results of this psychometric validation study support the validity and reliability of the previously developed electronic instrument for real-time GI symptom assessment in an IBS population, based on the experience sampling methodology. Moreover, this ESM-PROM has the advantage of a more precise and reliable assessment of individual symptom patterns over time, without the bias of peak reporting, compared with traditional end-of-day and end-of-week retrospective assessment methods. In addition, it offers possibilities to analyze symptom-symptom and symptom-environment interactions, which should be further evaluated in future studies. Altogether, this study highlights the potential of the ESM-PROM as a valuable tool for personalized healthcare in IBS to effectively monitor disease course and treatment response in both clinical and research settings for both patients and healthcare professionals.

## CONFLICTS OF INTEREST

**Guarantor of the article:** Daniel Keszthelyi, MD, PhD.

**Specific author contributions:** M.B. and L.V.: wrote the paper. M.B., L.V., J.S., R.L., and I.M.: collected data. L.V., D.J., Q.A., M.S., A.M., D.K., J.B., C.L., J.K., Z.M., S.K., H.B., E.Q., and D.K.: were involved in study design. M.B. and L.V.: analyzed data. D.J., Q.A., M.S., H.T., A.M., and D.K.: supervised the study. All authors contributed to revision of the manuscript and agreed to the submission of the article.

**Financial support:** The MEASuRE study was financially supported by a grant received from Grünenthal, GmbH (Aachen, Germany). The DISCOvERIE study was financially supported by the Horizon 2020 Framework Program (DISCOvERIE 848228). The ESM linaclotide study was financially supported by a grant received from AbbVie (North Chicago, IL).

**Potential competing interests:** M.B., L.V., J.S., R.T., Q.A., I.M., C.L., J.K., E.Q., S.K., and A.M. declare no conflicts of interests. D.J. has received research funding from public-private partnership grants of Top Knowledge Institute (TKI) Agri&Food and Health Holland, by the Carbokinetics program as part of the NWO-CCC Partnership Program, by Organic A2BV/Mothersfinest BV and by H2020 DISCOvERIE/848228 (outside the submitted paper). M.S. has received unrestricted research grants from Genetic Analysis AS and BioGaia, has participated as a Consultant/Advisory Board member of Danone Nutricia Research, Ironwood, Biocodex, Genetic Analysis AS, Tillotts, Takeda, Kyowa Kirin, AbbVie, BioGaia, and Cinclus Pharma, and received speaker's fee from Tillotts, Kyowa Kirin, Takeda, Biocodex, Sanofi, AbbVie, Janssen Immunology, Pfizer, Ferrer, BioGaia, Renapharma, Mayoly, and Bromatech. J.B. was an employee of Grunenthal GmbH until March 2022 and thereafter worked as an independent consultant in pharma under the umbrella of JBPharmaConsult. Z.M. has received research funding from ZonMw, MLDS, Niels Stensen Fellowship, TKI (private-public partnership), and Galapagos, participated in advisory boards of Johnson & Johnson, Lilly, Pfizer, and AbbVie, and received speaker's fee from Galapagos, Takeda, and Lilly (paid to host institute). H.T. has participated as a Consultant/Advisory Board member for Cinclus Pharma, Dr Falk Gmbh, PRO.MED.CS, and VIPUN and received speaker's fee from Tillotts, Takeda, and Shire. D.K. has received research funding from ZonMw, MLDS, UEG, Rome Foundation, Horizon Europe, Horizon 2020, Grunenthal, and Allergan and speaker's fee from Dr Falk (paid to host institute).

**Data transparency statement:** Individual participant data will not be available for sharing. Other document—i.e., study protocol, statistical analysis plan, and analytic code—will be available immediately after publication with no end date for researchers who provide a methodologically sound proposal with the purpose of achieving aims in the approved proposal. Proposals and requests should be directed to m.bosman@maastrichtuniversity.nl to gain access; data requestors will need to sign a data access agreement.

**Clinical trial registry:** Studies were registered in the US National Library of Medicine: NCT02880722 and NCT03336034.

**Study Highlights**

**WHAT IS KNOWN**

✓ Real-time symptom assessment in irritable bowel syndrome (IBS) using an experience sampling method (ESM)-based patient-reported outcome measure (PROM) is suggested to be more appropriate than currently used retrospective reports.

**WHAT IS NEW HERE**

✓ The ESM-PROM shows strong validity and reliability forgastrointestinal symptom assessment in IBS.
✓ The ESM-PROM enables detailed dynamic assessment of individual symptom patterns and triggers over time.
✓ The ESM-PROM is paving its way to be a valuable tool for monitoring disease and treatment response in personalized healthcare.
✓ The ESM-PROM offers insights into symptom-symptom and symptom-environment interactions, aiding IBS pathophysiology research and future interventions.

## Supplementary Material
