# Supplementary material for: Results From a Psychometric Validation Study: Patients With Irritable Bowel Syndrome Report Higher Symptom Burden Using End-of-Day Vs Real-Time Assessment
Source: Am J Gastroenterol. 2024 Sep 23;120(5):1098–107. doi: 10.14309/ajg.0000000000003091 (PMC12043265; doi:10.14309/ajg.0000000000003091)
Supplement: Supplementary file 1 [file acg-120-1098-s001.docx]

**Results from a psychometric validation study: irritable bowel syndrome patients report higher symptom burden using end-of-day versus real-time assessment**

**Michelle Bosman, MD^1*^, Lisa Vork, MD, PhD^1*^,** Daisy Jonkers, PhD^1^, Johanna Snijkers, MD^1^, Rabia Topan, MBChB, BSc, MRCP^2^, Qasim Aziz, FRCP, PhD^2^, Irina Midenfjord, PhD^3^, Magnus Simren, MD, PhD^3^, Ad Masclee, MD, PhD^1^, ESM study group**, Daniel Keszthelyi, MD, PhD^1^.

* Michelle Bosman and Lisa Vork Author share co-first authorship.

** ESM study group: John Bothmer^4^, Carsten Leue^5^, Joanna Kruimel^1^, Zlatan Mujagic^1^, Sander van Kuijk^6^, Hans Tornblom^3^, Emilio Quetglas^4^.

**Supplementary Material**

**Supplementary Appendix 1: detailed information of the three studies**

Cohort 1 (MEASuRE study): 150 IBS patients aged between 18-70 years were included between 2017 and 2021 in this multicenter, prospective, cross-sectional study focused on validation of ESM-PROM and its evaluation of triggers for chronic abdominal pain. The current manuscript presents data as a part of this project. The study was performed in three secondary/tertiary European hospitals: Maastricht UMC+, the Netherlands; Sahlgrenska University Hospital, Sweden; Royal London Hospital, United Kingdom. Patients were primarily recruited via the outpatient clinics of the above-mentioned hospitals, besides recruitment of patients that previously participated in the Maastricht IBS cohort (Weerts et al., 2019. Neurogastroenterol Motil). All patients met the Rome IV criteria for IBS, which were confirmed in a face-to-face interview by the investigator during the screening visit. No alarm symptoms or organic causes for symptoms were present and patients were excluded if they had abdominal surgery in the past (except for uncomplicated appendectomy, cholecystectomy, and/or hysterectomy) or if they changed medication use within one month before the start of the study until the end of the study. All study participants could only participate if they could understand the Dutch, Swedish, or English language, respectively, and were able to use the smartphone application. Participation in the study involved the completion of the ESM assessment for 7 days, the end-of-day diaries for 7 days, and the end-of-week questionnaires.

Cohort 1 (Linaclotide study): 12 IBS-C patients aged between 18-75 years were included between 2018 and 2021 in a prospective, observational, single-group, open-label study assessing responsiveness to linaclotide with the ESM-PROM in IBS-C. Only the baseline data from this study were included in the current manuscript. The study was performed at the outpatient clinic of the secondary/tertiary hospital Maastricht UMC+, the Netherlands. All patients diagnosed with Rome IV IBS-C who were prescribed linaclotide 290 microgram once daily by their treating physician were approached prior to starting the therapy. In- and exclusion criteria are similar to the MEASuRE study, in addition to the exclusion of patients that have a history of laxative abuse and current use of medications that could cause constipation. Data collection was identical to the MEASURE study.

Cohort 2 (DISCOvERIE study): 68 IBS patients aged 18 years and older were included between June 2021 and December 2022 in the DISCOvERIE European multi-center prospective, longitudinal, case-control observational study aimed at enhancing the understanding on IBS and its comorbidities and risk factors. The current manuscript presents data of one part of this study in one secondary/tertiary center, Maastricht UMC+, the Netherlands. Patients’ recruitment occurred through the outpatient clinics of Maastricht UMC+, self-referral via public advertisements, social media, and the Dutch IBS patient federation, as well as via other IBS studies conducted in Maastricht. In- and exclusion criteria are similar to the MEASURE study, with the exception that all medication use was permitted. Participation in the study involved the completion of questionnaires assessing clinical characteristics, the collection of biological samples, stress function assessments, and the completion of e-Health measurements, which included ESM-PROM and wearable data collection, at baseline, after 1 year, and after 2 years. This manuscript presents data on the e-Health measurement part of the study at baseline, *i.e.,* the completion of the ESM assessment for 7 days and the end-of-week questionnaires.

**Supplementary Appendix 2: detailed information of the questionnaires**

Severity of IBS symptoms was assessed using the Gastrointestinal Symptom Rating Scale for Irritable Bowel Syndrome (GSRS-IBS) and the IBS Severity Scoring System (IBS-SSS).

In the GSRS-IBS^21^, patients are asked to rate 13-items regarding their abdominal symptoms (composes sub-scores for e.g., abdominal pain, abdominal bloating, constipation, diarrhea, satiety) on a 1-7 scale, with a higher total score indicating more severe symptom with a recall period of one week. GSRS-IBS was completed in cohort 1.

In the IBS-SSS^22^, patients are asked to rate 5-items regarding their abdominal symptoms (*i.e.,* frequency of pain, severity of pain, severity of distention, bowel habit dissatisfaction, and daily life interference) on a visual analog scale (VAS) from 0 to 100 with a recall period of 10 days. Higher scores indicate more severe symptoms. Consequently, patients were divided into three groups based on the severity scores: mild (<175), moderate (175–300) or severe (>300) IBS symptoms. IBS-SSS was completed in all participants.

Comorbid symptoms of anxiety and depression were assessed using both the Generalized Anxiety Disorder-7 (GAD-7) and the Patient Health Questionnaire-9 (PHQ-9), respectively. In the GAD-7^23^, patients are asked to rate 7-items regarding worry and anxiety symptoms on a 4-point response scale, with a higher total score indicating more severe anxiety symptoms. Consequently, the patients were divided into four groups based on the severity scores: none-minimal (0–4), mild (5–9), moderate (10–14), and severe (15-21) symptom severity. In the PHQ-9^24^, patients are asked to rate 9-items regarding depressive symptoms on a 4-point response scale, with a higher total score indicating more severe depressive symptoms. Consequently, the patients were divided into five groups based on the severity scores: none-minimal (0–4), mild (5–9), moderate (10–14), moderately-severe (15-19), and severe (20-27) symptom severity. The GAD-7 and PHQ-9 were completed in all participants.

**Supplementary Appendix 3: detailed information of the statistical analysis**

All analyses were performed using R version 4.2.2 (R Core Team (2022). R: A language and environment for statistical computing. R Foundation for Statistical Computing, Vienna, Austria.). Participants who completed at least 23 out of 70 assessments on ESM were included in the analysis; 11 participants were excluded for not meeting this completion rate.^25, 26^ Baseline characteristics are presented as mean with corresponding standard deviation or number of totals with proportion (%), based on non-missing observations. Differences in baseline characteristics between the two cohorts were tested with an analysis of variance (ANOVA) with post-hoc Bonferroni correction (continuous variables) or χ^2^-test (categorical variables). In addition, correction for multiple testing was applied using the Bonferroni correction (p ≤ 0.007 was considered statistically significant).

Concurrent validity was assessed by comparing ESM scores to end-of-day diary scores (*i.e.,* on day-level; no repeated measures within the day) in cohort 1, and to end-of-week questionnaire scores (*i.e.,* on week-level; no repeated measures within the week) in all participants. To compare ESM scores with end-of-day diary scores, the maximum and mean scores for ESM were calculated for each of the seven days (*i.e.,* combining all repeated measurements of all participants for the concerning day). GI symptoms scored in both ESM and the end-of-day diary were abdominal pain, belching, bloating, flatulence, and urge to defecate in the MEASuRE study; and abdominal pain, bloating, and urge to defecate in linaclotide study. Associations between end-of-day scores and mean ESM scores were tested using linear mixed-effects models with the end-of-day score as the dependent and the mean ESM score as the independent variable, a random intercept, and correcting for repeated measures by using an autoregression (AR1) covariate structure. The level of agreement between end-of-day and mean ESM scores was evaluated by calculating intra-class correlation coefficients (ICC), based on a single-rating, consistency, two-way model. In addition, differences between the end-of-day and mean/max ESM scores were tested using intercept-only linear mixed-effects models with the delta scores (*i.e.,* difference between end-of-day diary and ESM) as the dependent variable and correcting for repeated measures by using an autoregression (AR1) covariate structure. ICC values between 0.5-0.75 are considered moderate and values above 0.75 good.^27, 28^

To compare ESM scores with end-of-week questionnaire scores, all ESM measures were averaged to one score per participant. Differences between the measurement methods were tested using the paired samples t-test and Pearson correlations were calculated. In order to harmonize the scores of ESM and GSRS, the ESM scores were rescaled from an 11-point to a 7-point NRS. For the comparison between ESM and IBS-SSS, the IBS-SSS scores were divided by 10; ESM scores were not rescaled. Differences between the answering scales from the PHQ-9 and GAD-7 versus ESM did not allow harmonization of the scores, so mean scores could not be compared between these questionnaires and ESM. Pearson correlation coefficients between 0.40-0.69 are considered moderate and values of 0.70 and above strong.^29^

Internal consistency and test-retest reliability was assessed in all participants. The internal consistency of the ESM-PROM was evaluated by dividing the items into five domains and calculating the Cronbach’s alpha coefficient per domain. Cronbach’s alpha values of 0.70-0.90 are considered to reflect good internal consistency.^28^

In order to assess test-retest reliability of the ESM-PROM we assumed that ESM scores during the first half-week of the study period (*i.e.,* days 1, 2, and 3) would show moderate-to-good consistency with the scores during the second half-week (*i.e.,* days 5, 6, and 7). For each participant and for each symptom a mean score for these two time-periods was calculated. The difference between these was tested using a paired samples t-test to exclude a time effect, and an ICC between the time-periods was calculated to assess agreement, using a two-way model based on average-measures and absolute agreement. ICC values between 0.75-0.90 are considered moderate and values greater than 0.90 excellent.^27, 28^

**Supplementary Figures**

**Supplementary Figure 1. Framework of the previously developed ESM-PROM, consisting of 25 items (in the figure summarized in 10 constructs) based on five domains**

**
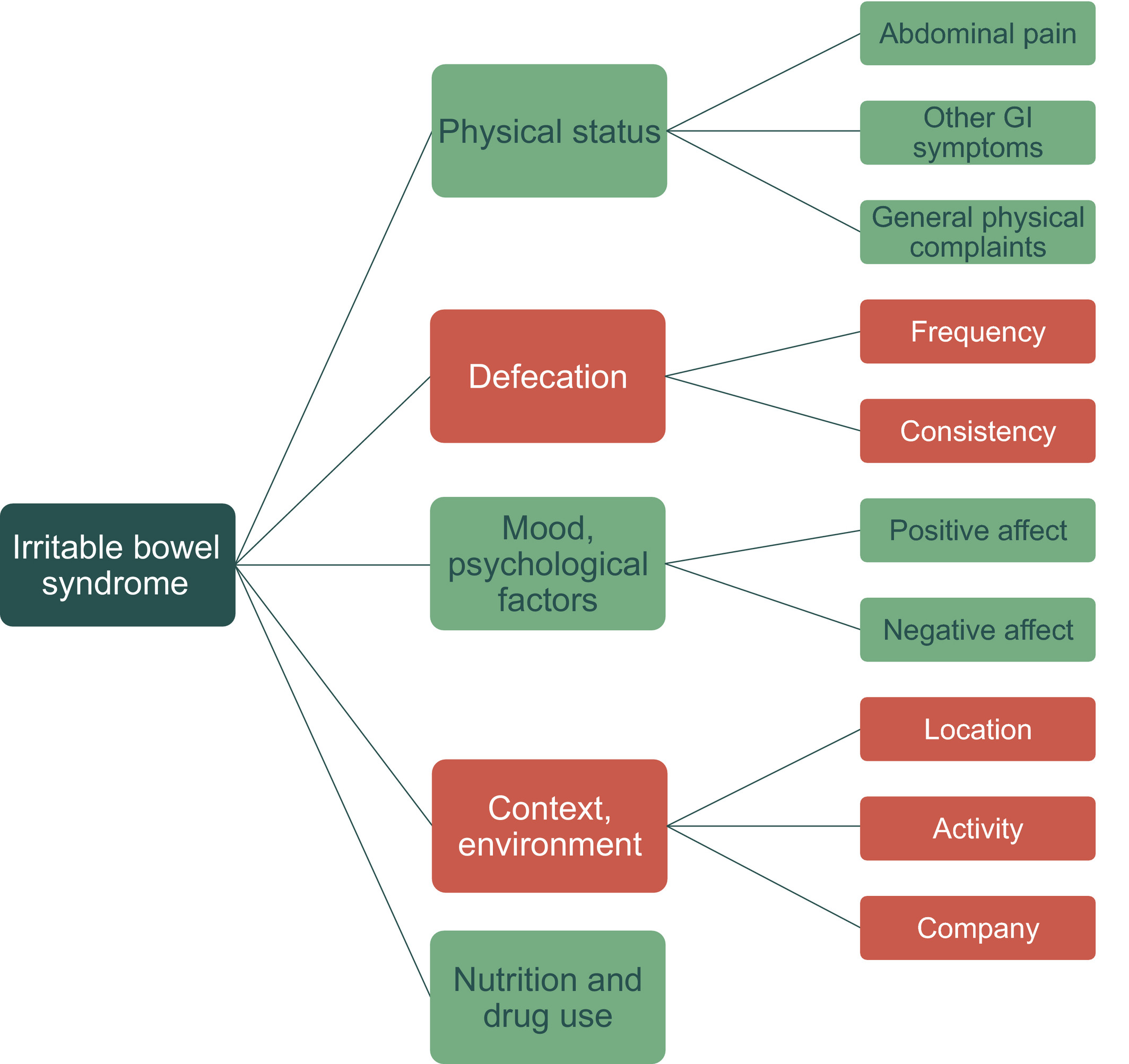
**

**Supplementary Figure 2. Completion rate with ESM; represented by number of participants (y-axis) per category of completed number of assessments (x-axis)** *(0-30 assessments: 13 participants, 31-40 assessments: 41 participants, 41-50 assessments: 52 participants, 51-60 assessments: 88 participants, 61-70 assessments: 36 participants).*

**Supplementary Figure 3. Abdominal pain scores (on an 11-point NRS) for ESM and end-of-day diary over the 7-day study period, for one IBS patient. Each day, 10 assessments were available for ESM; one assessment was completed at the end of each day in the diary.** *This is figure shows fluctuating ESM abdominal pain scores, capturing moments without pain and moderate pain, while the end-of-day diary consistently records moderate-to-high pain scores daily, emphasizing the notable disparity between the two assessment methods.*

***
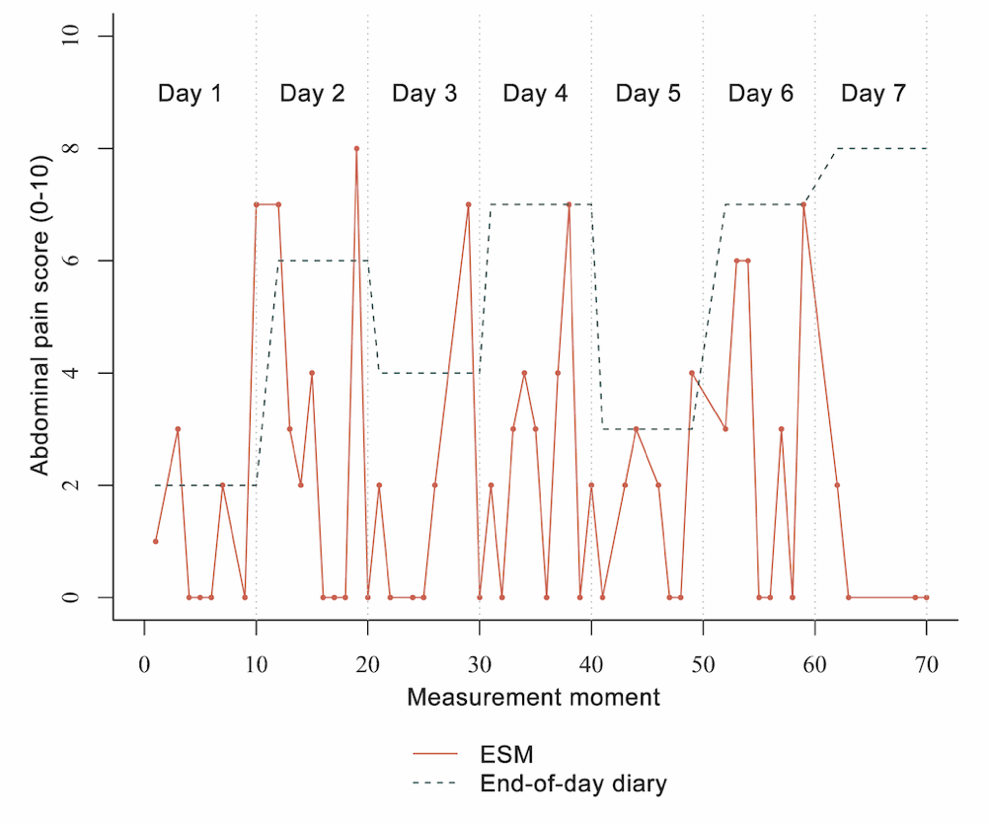
***

**Supplementary Tables**

| Supplementary Table 1. Summary of patient demographic and characteristics, divided for each country. | | | | | |
| --- | --- | --- | --- | --- | --- |
|  | **Total** | **The Netherlands** | **United Kingdom** | **Sweden** |  |
|  | ***N* = 230** | ***N* = 117** | ***N* = 79** | ***N* = 34** | **p-value** |
| Age, years  Mean (95% CI)  Range | 41.2 (39.3, 43.1)  19-75 | 43.7 (40.9, 46.5)  19-75 | 37.2 (34.2, 40.1)  20-68 | 41.9 (37.8, 46.0)  23-71 | **0.006** |
| Sex, n (%)  Female | 185 (80.4) | 89 (76.1) | 70 (88.6) | 26 (76.5) | 0.078 |
| BMI, mean (95% CI) | 25.0 (24.3, 25.8) | 25.3 (24.2, 26.3) | 25.6 (24.0, 27.1) | 23.1 (21.7, 24.4) | 0.100 |
| IBS subtype, n (%)  Diarrhea  Constipation  Mixed  Undefined | 108 (47.0)  66 (28.7)  42 (18.3)  14 (6.1) | 48 (41.0)  39 (33.3)  23 (19.7)  7 (6.0) | 41 (51.9)  19 (24.1)  12 (15.2)  7 (8.7) | 19 (55.9)  8 (23.5)  7 (20.6)  0 (0) | 0.289 |
| IBS symptom severity, n (%) on IBS-SSS (n=222)  In remission  Mild  Moderate  Severe | 4 (1.8)  24 (10.8)  106 (47.8)  88 (39.6) | 2 (1.8)  15 (13.4)  57 (50.9)  38 (33.9) | 1 (1.3)  8 (10.3)  37 (47.4)  32 (41.0) | 1 (3.1)  1 (3.1)  12 (37.5)  18 (56.3) | 0.320 |
| Educational level (n=225)  University degree | 89 (39.6) | 24 (20.7) | 47 (60.3) | 18 (58.1) | **<0.001** |
| Employment status (n=225)  Currently studying  Employed  Unemployed  Incapacitated for work  Homemaker  Retired | 22 (9.8)  155 (68.9)  3 (1.3)  23 (10.2)  5 (2.2)  17 (7.6) | 12 (10.3)  72 (62.1)  0 (0)  17 (14.7)  2 (1.7)  13 (11.2) | 7 (9.0)  58 (74.4)  2 (2.6)  5 (6.4)  3 (3.9)  3 (3.9) | 3 (9.7)  25 (80.7)  1 (3.2)  1 (3.2)  0 (0)  1 (3.2) | 0.102 |
| Bold indicates significant difference between the cohorts (p≤0.007). For definition of subgroups based on severity scores see supplementary information 2. Educational level: university degree versus no university degree. CI=confidence interval; N=number; BMI=body mass index (kg/m^-2^); IBS=irritable bowel syndrome; IBS-SSS=IBS Severity Scoring System. | | | | | |

| **Supplementary Table 2. Mean numbers of completed ESM assessments per study day (out of a total of 10 assessments each day), averaged for all participants (*N* = 230).** | |
| --- | --- |
| **Study day** | **Mean numbers of completed ESM assessments**  Mean ± SD |
| **1** | 7.65 ± 1.66 |
| **2** | 7.89 ± 1.55 |
| **3** | 7.80 ± 1.69 |
| **4** | 7.61 ± 1.69 |
| **5** | 7.68 ± 1.73 |
| **6** | 7.47 ± 1.81 |
| **7** | 7.51 ± 1.84 |
| ESM=experience sampling method. | |

| **Supplementary Table 3. Mean numbers of completed ESM assessments per weekday (out of a total of 10 assessments each day), averaged for all participants (*N* = 230).** | |
| --- | --- |
| **Study day** | **Mean numbers of completed ESM assessments**  Mean ± SD |
| **Monday** | 7.85 ± 1.70 |
| **Tuesday** | 7.54 ± 1.63 |
| **Wednesday** | 7.64 ± 1.69 |
| **Thursday** | 7.63 ± 1.69 |
| **Friday** | 7.79 ± 1,70 |
| **Saturday** | 7.53 ± 1.75 |
| **Sunday** | 7.65 ± 1.82 |
| Participants did not start on a standard weekday; this is different for each participant. ESM=experience sampling method. | |

| **Supplementary Table 4. Mean numbers of completed ESM assessments per timepoint each day (out of a total of 7 completed timepoints in 7 days), averaged for all participants (*N* = 230).** | |
| --- | --- |
| **Timepoint each day** | **Mean numbers of completed ESM assessments**  Mean ± SD |
| **1** | 3.77 ± 2.07 |
| **2** | 4.81 ± 1.60 |
| **3** | 5.09 ± 1.41 |
| **4** | 5.08 ± 1.45 |
| **5** | 5.09 ± 1.39 |
| **6** | 5.04 ± 1.40 |
| **7** | 5.09 ± 1.51 |
| **8** | 5.13 ± 1.51 |
| **9** | 5.19 ± 1.45 |
| **10** | 5.07 ± 1.59 |
| Timepoint 1 indicating the first assessment of the day (morning) and 10 the last assessment of the day (evening). ESM=experience sampling method; SD=standard deviation. | |
